# Supplementary material for: Resistance to Plant Parasites in Tomato Is Induced by Soil Enrichment with Specific Bacterial and Fungal Rhizosphere Microbiome
Source: Int J Mol Sci. 2023 Oct 21;24(20):15416. doi: 10.3390/ijms242015416 (PMC10607013; doi:10.3390/ijms242015416)
Supplement: Supplementary file 1 [file ijms-24-15416-s001.zip › ijms-2661307-supplementary.pdf]

Table S1. Plant height (PH), number of egg masses per root system (EMs), and female fecundity (FF) of tomato plants harvested 2 months after inoculation with the root-knot nematode *M. incognita*. Treatments with 0.25 g Myco and 0.08 g Ozor g^{-1} plant fresh weight were carried out 5 days before nematode inoculation. Means ± standard deviations for treated (Myco, Ozor) and untreated (contr) plants were separated by a paired *t*-test (*P<0.05). The percentages of increase/decrease of significantly different factors are in parentheses.

	**contr**	**Myco**	**contr**	**Ozor**
PH	70±19	65±20	76±12	81±13
EMs	94±45	64±31*(-32)	88±44	59±16*(-33)
FF	203±77	432±113*(113)	214±86	392±217*(83)

Table S2. Tomato plants were soil-drenched with liquid suspensions (0.5, 1.0, 2.0 10^8 CFU/plant) of *Bacillus subtilis* (Bt) and *Pseudomonas fluorescens* ATCC 13525 (Pf); other plants were soil-drenched with the suspensions used to grow the bacteria and considered as the controls (Cntr). All plants were inoculated with M. incognita after 5 days. Two months after nematode inoculation, growth factors, such as plant height (PH), and infection factors, such as the number of egg masses per root system (EMs) and female fecundity (FF), were detected. Means ± standard deviations for treated (Bt/Pf +0.5, +1.0, +2.0) and untreated (Cntr) plants were separated by a paired t-test (*P<0.05). The percentages of increase/decrease of significantly different factors are in parentheses.

	**Cntr**	**+ 0.5 Pf**	**+ 1.0 Pf**	**+ 2.0 Pf**
PH	77±11	86±18	71±13	78±13
EMs	56±39	64±19	59±25	62±22
FF	283±54	376±140*(33)	213±48*(-25)	411±200*(45)

	**Cntr**	**+ 0.5 Bt**	**+ 1.0 Bt**	**+ 2.0 Bt**
PH	61±7	76±12*(24)	71±10	76±7*(24)
EMs	45±20	81±31*(79)	88±32*(95)	76±36*(68)
FF	526±96	447±78	386±93*(-27)	458±147
